# Supplementary material for: α3 Chains of type V collagen regulate breast tumour growth via glypican-1
Source: Nat Commun. 2017 Jan 19;8:14351. doi: 10.1038/ncomms14351 (PMC5253704; doi:10.1038/ncomms14351)
Supplement: Supplementary Information — Supplementary Figures and Supplementary Tables [file ncomms14351-s1.pdf]

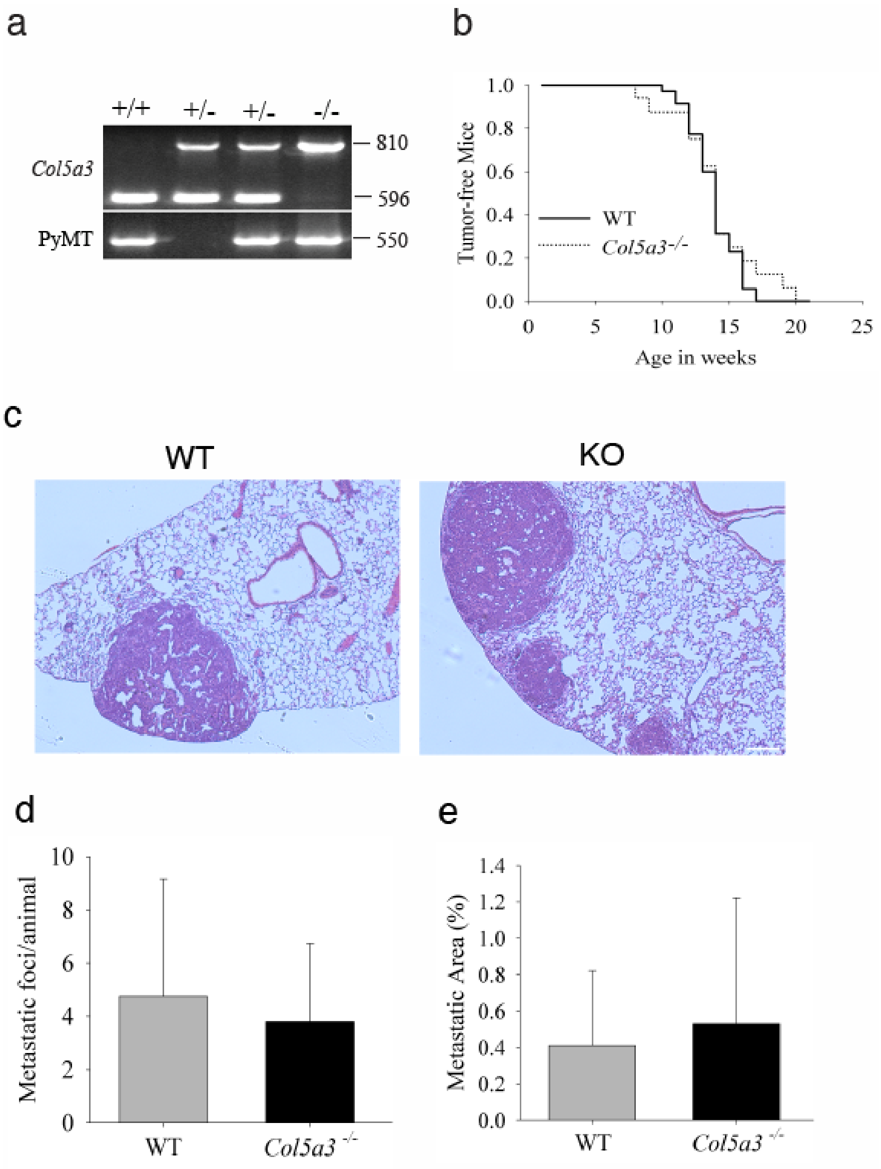

Supplementary Figure 1. **Genotyping, tumor latency, and lung metastases of F2 KO/PyMT and WT/PyMT mice obtained from *Col5α3*$^{-/-}$-MMTV-PyMT crosses.** (**a**) PCR genotyping of ear punch genomic DNA from progeny of intercrosses of *Col5α3*$^{+/-}$ and *Col5α3*+/- with/without the MMTV-PyMT oncogene. 810, 596, and 550 bp bands correspond to *Col5α3* null alleles and WT alleles, or the MMTV-PyMT transgene, respectively. (**b**) Kaplan-Meir plots of WT/PyMT (WT, n=34) and KO/PyMT (*Col5α3*$^{-/-}$, n=23) mice. (**c**) H&E stained sections of WT/PyMT and KO/PyMT lungs with representative metastatic foci are shown. Quantitation shows no significant differences in numbers of metastatic foci (**d**) or total metastatic area (**e**) between 20 weeks old WT/PyMT (n=14) and KO/PyMT (n=11) lungs. Data are presented as mean ± SD. Statistical analysis - 2-tailed Student's *t* test (differences considered significant at $P < 0.05$). Also, no significant differences in numbers of metastatic foci were found between lungs of WT/PyMT and KO/PyMT (n=8 each) mice sacrificed when total tumor volume reached 10% of body weight.

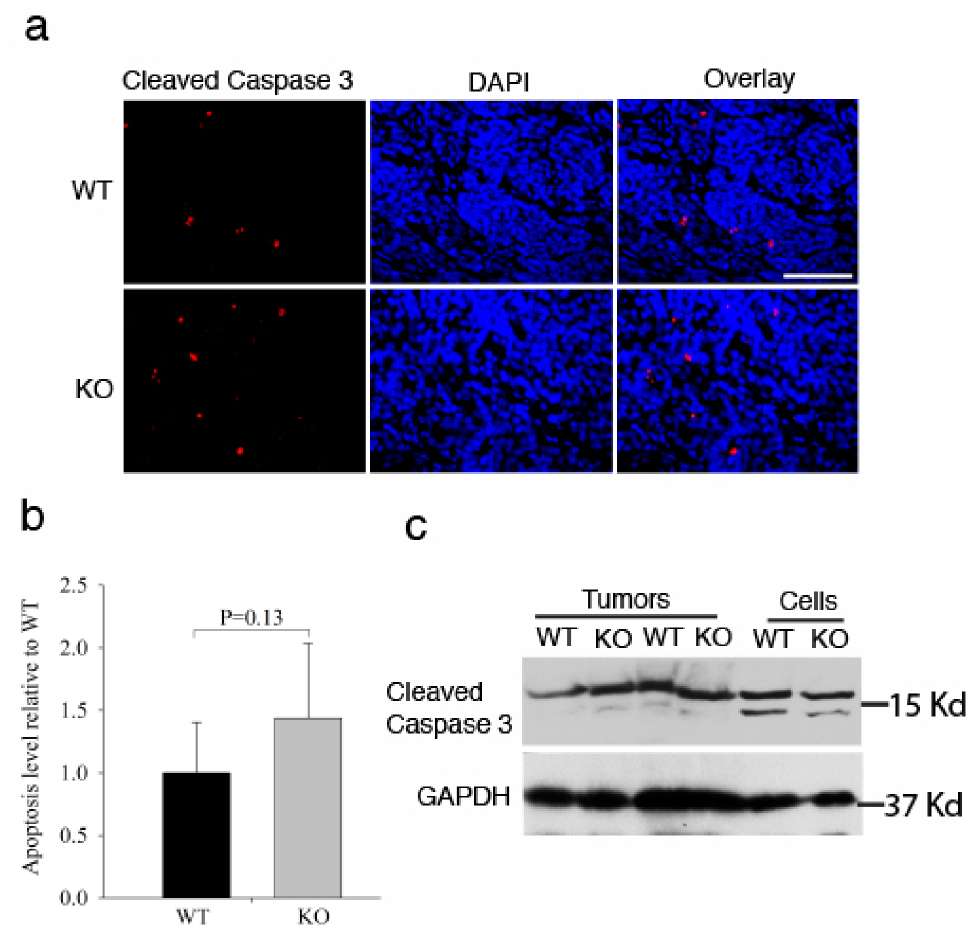

Supplementary Figure 2. **No apparent difference in apoptotic indices in KO/PyMT and WT/PyMT tumors.** (**a**) Immunofluorescent staining of tumors was performed with antibodies to cleaved caspase 3. DAPI staining marks nuclei (blue). White scale bar, 50 μm. (**b**) To quantitate the results from immunofluorescent staining, cleaved caspase 3-positive cells were counted in 3 different frames from a tumor from each of 5 different KO/PyMT or WT/PyMT mice. Positive cell numbers were then averaged for the KO/PyMT and WT/PyMT tumors. Data are presented as mean ± SD. Statistical analysis was via 2-tailed Student's *t* test, with differences considered significant at $P < 0.05$. (**c**) Immunoblotting of the extracts of WT/PyMT and KO/PyMT tumors and cultured primary tumor cells was performed using antibodies to cleaved caspase 3 and to GAPDH, the latter as a loading control.

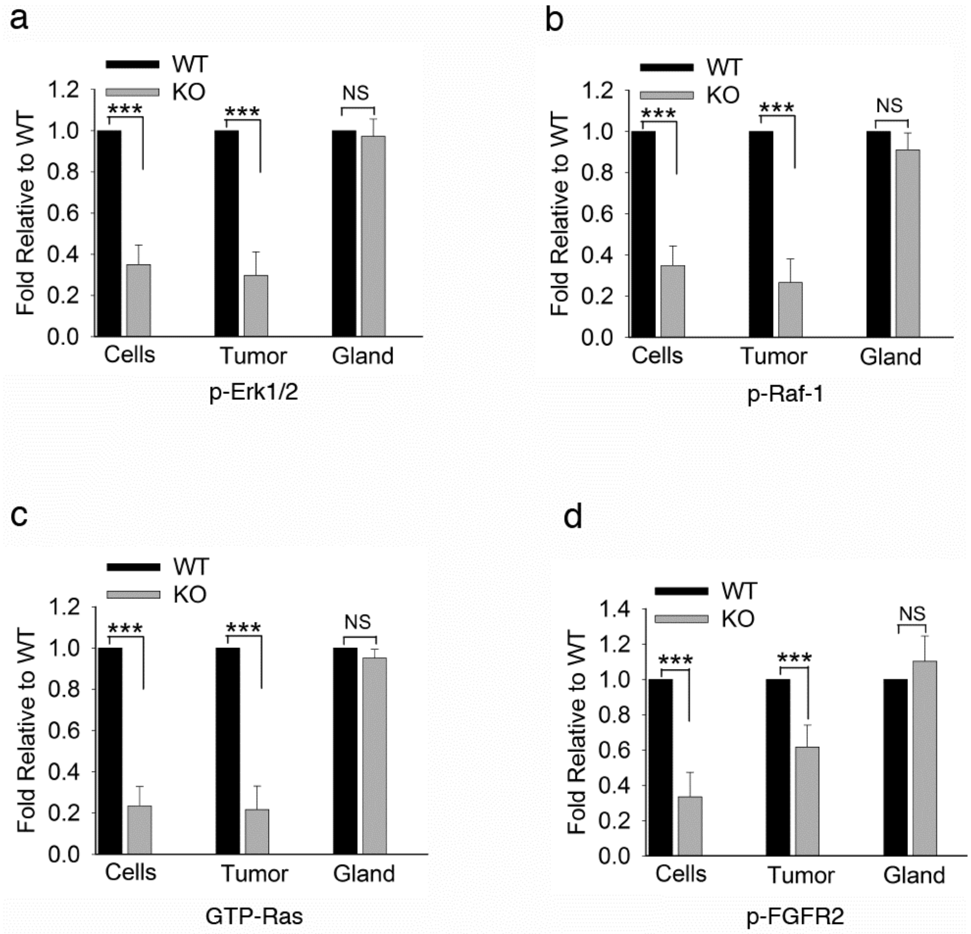

Supplementary Figure 3. **KO/PyMT tumor cells have significantly reduced FGF/Ras/Raf/ERK signaling, compared to WT/PyMT controls**. Immunoblots of Fig. 3 **g** and **h** were quantified via scanning of films and ImageJ for p-Erk1/2 (**a**), p-Raf-1 (**b**), GTP-Ras (**c**); or via use of a LI-COR Odyssey Fc imager for p-FGFR2 (**d**). Immunoblots for each protein antigen were repeated 3 times, from 3 independent tumor or cell lysates. For histograms, the mean intensity of WT/PyMT sample bands was given an arbitrary value of 1, and the fold increase/decrease in intensities of corresponding KO/PyMT bands is given relative to the WT/PyMT value of 1. Data are presented as mean ± SD. ***, $P<0.005$. Statistical analysis was via 2-tailed Student's *t* test, with differences considered significant at $P < 0.05$. There were no significant differences for levels of total Raf-1, Ras, Erk1/2, or FGFR2.

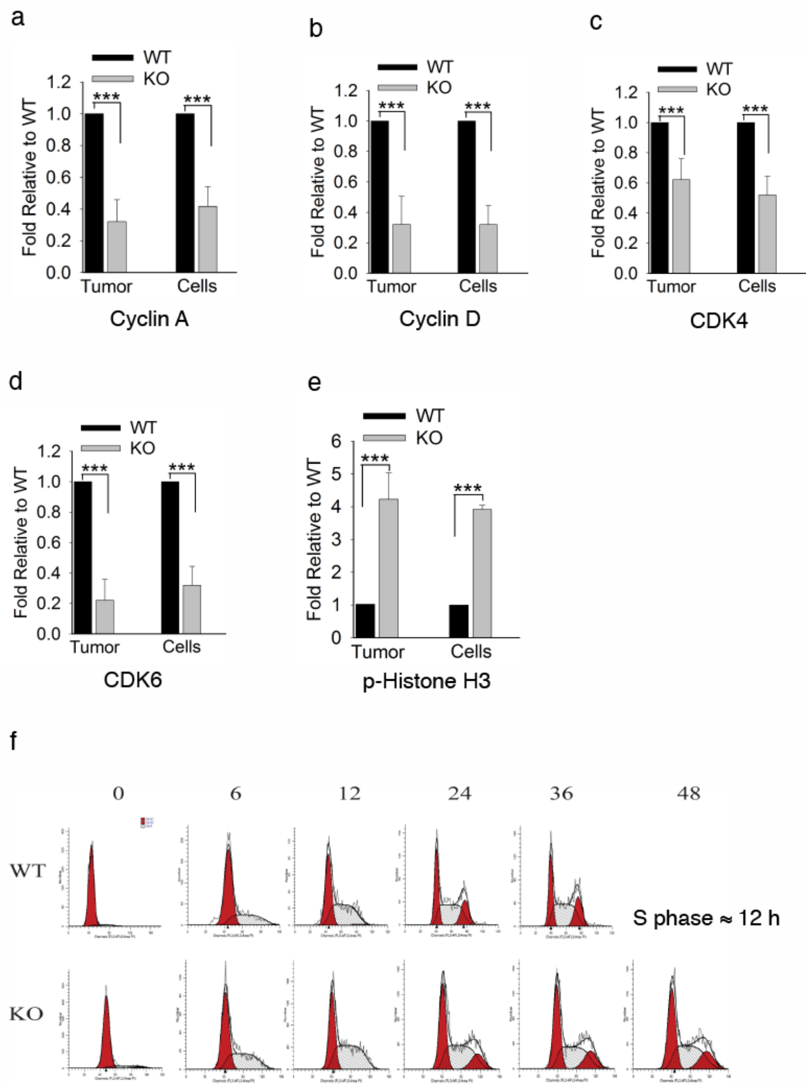

Supplementary Figure 4. **Quantification of Cyclin, CDK, and p-histone H3 differences between WT/PyMT and KO/Py/MT tumors and tumor cells; similar lengths of S-phase in KO/PyMT and WT/PyMT tumor cells.** Immunoblots of Fig. 4 **c** and **f** were quantified by scanning of films and ImageJ for Cyclin A (**a**), Cyclin D (**b**), CDK4 (**c**), CDK6 (**d**), or p-H3 (**e**). Blots for each protein were repeated 3 times, from 3 independent tumor/cell lysates. For histograms, the mean intensity of WT/PyMT sample bands was given a value of 1, and the fold increase or decrease in corresponding KO/PyMT band intensity is given relative to the WT /PyMT value of 1. Data are presented as mean ± SD. ***; $P<0.005$. Statistical analysis was via 2-tailed Student's *t* test, with differences considered significant at $P < 0.05$. There were no significant differences for Cyclin E or Cyclin B levels. (**f**) Cells were grown in growth medium, then stained with propidium iodide and analyzed by flow cytometry. Analysis at indicated time points of G1-sorted cells released into growth medium found no difference in WT/PyMT and KO/PyMT S-phase length (~12 h).

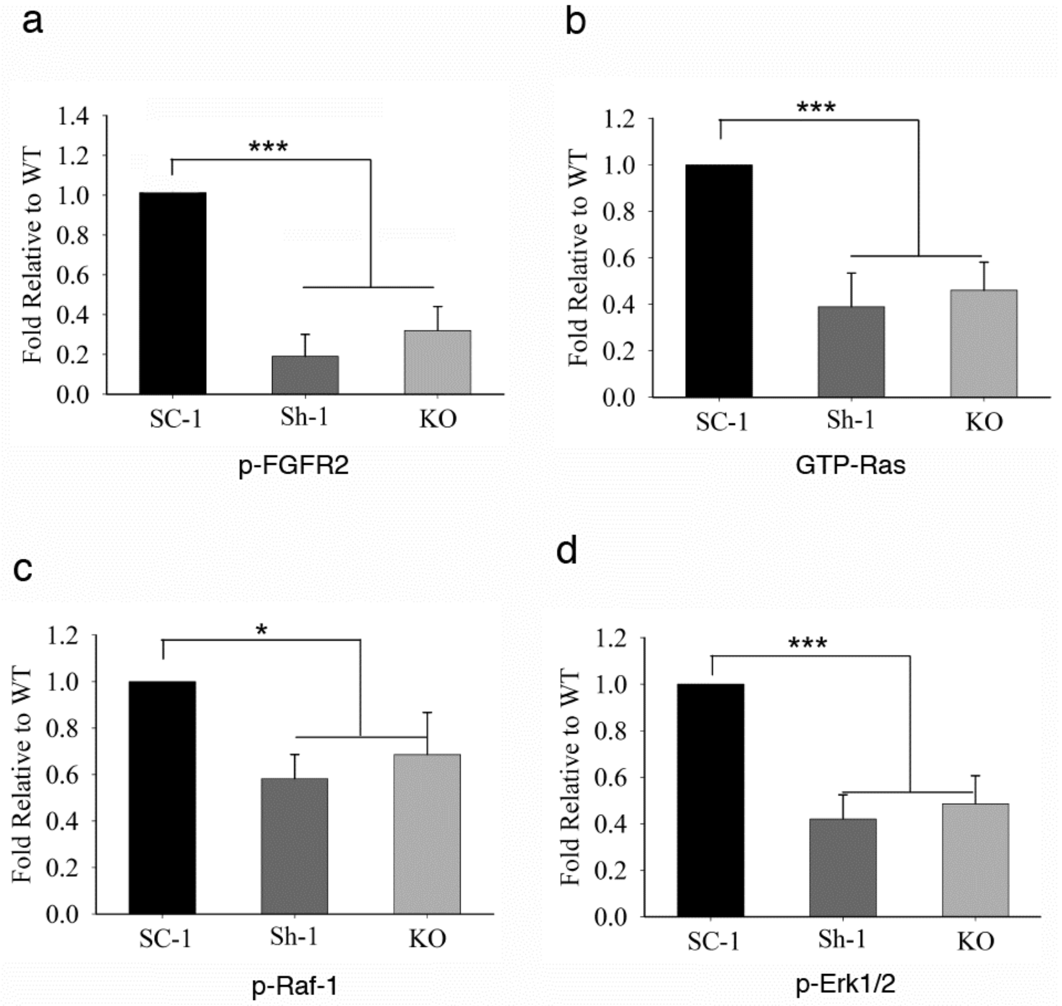

Supplementary Figure 5. **Significantly reduced FGF/Ras/Raf/ERK signaling upon shRNA knockdown of GPC1.** Immunoblots of Figure 6 **b** and **c** were quantified via use of a LI-COR Odyssey Fc imager for p-FGFR2 (**a**), GTP-Ras (**b**), p-Raf (**c**); or p-Erk1/2 (**d**) in KO/PyMT tumor cells, or in WT/PyMT tumor cells infected with adenoviral vector Ad-Sh-1, for GPC1 knockdown, or with scrambled control vector Ad-Sc-1. Blots for each protein were repeated 3 times, from 3 independent cell lysates. For histograms, the mean intensity of bands from lysates of Sc-1-treated WT/PyMT cells was given an arbitrary value of 1, and fold increase/decrease in intensities of corresponding bands from lysates of KO/PyMT or Sh-1-treated WT/PyMT cells is given relative to the WT/PyMT value of 1. Data are presented as mean ± SD. *, $P<0.05$; ***, $P<0.005$. Statistical analysis was via 2-tailed Student's *t* test, with differences considered significant at $P < 0.05$. There were no significant differences for levels of total Raf-1, Erk1/2, or FGFR2.

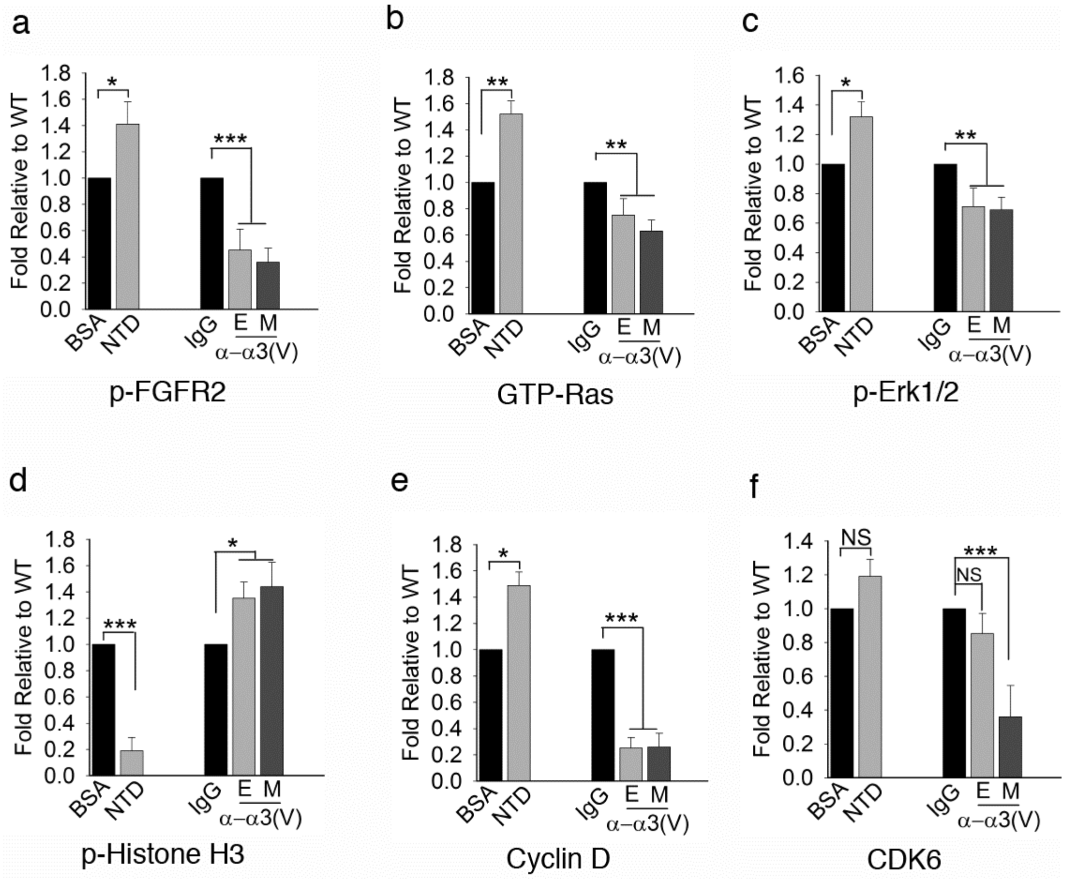

Supplementary Figure 6. **Quantification of effects of treatment with recombinant α3(V)-NTD sequences and anti-α3(V)-NTD antibodies on levels of FGF/Ras/ERK signaling, phospho-histone H3, Cyclin D, and CDK6**. Immunoblots of Figure 8 b and d were quantified via LI-COR Odyssey Fc imager, for p-FGFR2 (**a**), GTP-Ras (**b**), p-Erk1/2 (**c**); or via scanning of films and ImageJ for p-Histone H3 (**d**), cyclin D (**e**), and CDK6 (**f**). There were no significant differences for levels of total Ras, Erk1/2, FGFR2, or histone H3 (not shown). Immunoblots for each protein antigen were repeated 3 times, from 3 independent cell lysates. For histograms, the mean intensity of bands from lysates of cells not treated with NTD or anti-α3(V) antibody was given an arbitrary value of 1, and the fold increase/decrease in intensities of corresponding bands from cells treated with NTD or anti-α3(V) antibody is given relative to that value of 1. Data are presented as mean ± SD. *, $P<0.05$; **, $P<0.01$; ***, $P<0.005$. Statistical analysis was via 2-tailed Student's *t* test, with differences considered significant at $P < 0.05$. In panel **f**, although CDK6 levels increase upon treatment of cells with NTD and decrease upon treatment of cells with antibodies raised against recombinant NTD sequences prepared in *E. coli* (E), which did not work as well as did the "M" antibodies raised against NTD sequences prepared in mammalian cells (see panel **f** and also Fig. 8 in the paper), these changes did not reach significance (NS) over the course of the three immunoblots examined, although they trended towards significance ($P<0.058$ and $P<0.065$, respectively).

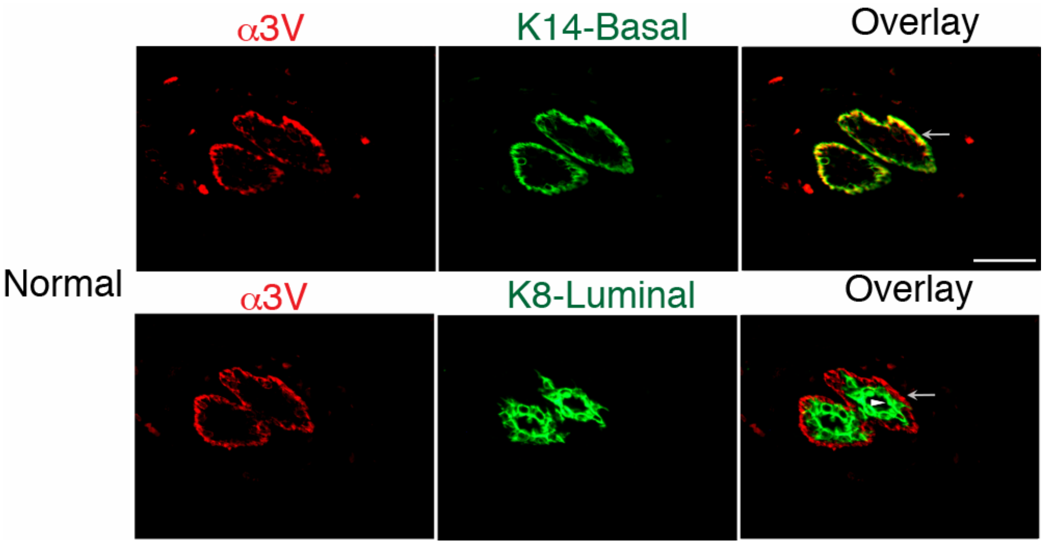

Supplementary Figure 7. α3(V) chains are associated with K14-positive basal cells and GPC1 is associated with K8-positive luminal cells in normal human mammary glands. Immunofluorescent staining was performed with antibodies to α3(V) chains (red), or to basal cell marker K14 (top panel, green), or luminal cell marker K8 (bottom panel, green). Overlay panels show areas of α3(V) and K14 co-localization (top panel, yellow), and the absence of co-localization of α3(V) and K8 (bottom panel). Arrowheads and arrows denote luminal and basal cells, respectively. White scale bar, 50 μm.

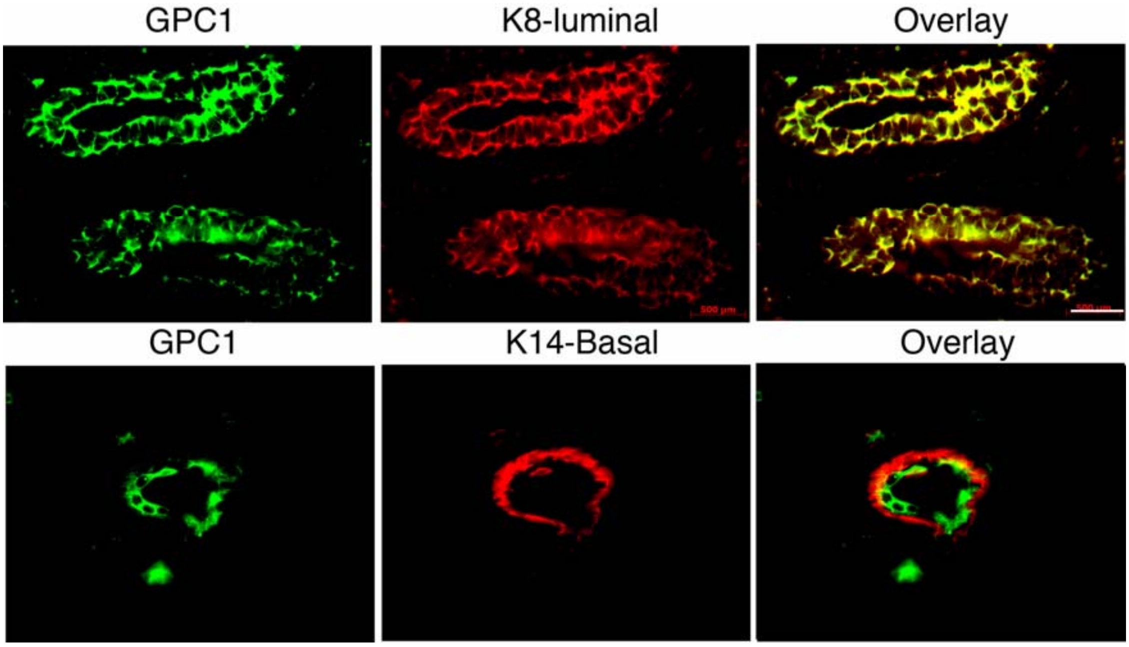

Supplementary Figure 8. GPC1 is associated with luminal, but not basal, cells in normal mouse mammary gland. Immunofluorescence co-localizaiton of GPC1 (green) with marker K8 (red) in luminal cells (top panels), but not with maker K14 (red, bottom panels). White scale bar, 50 μm.

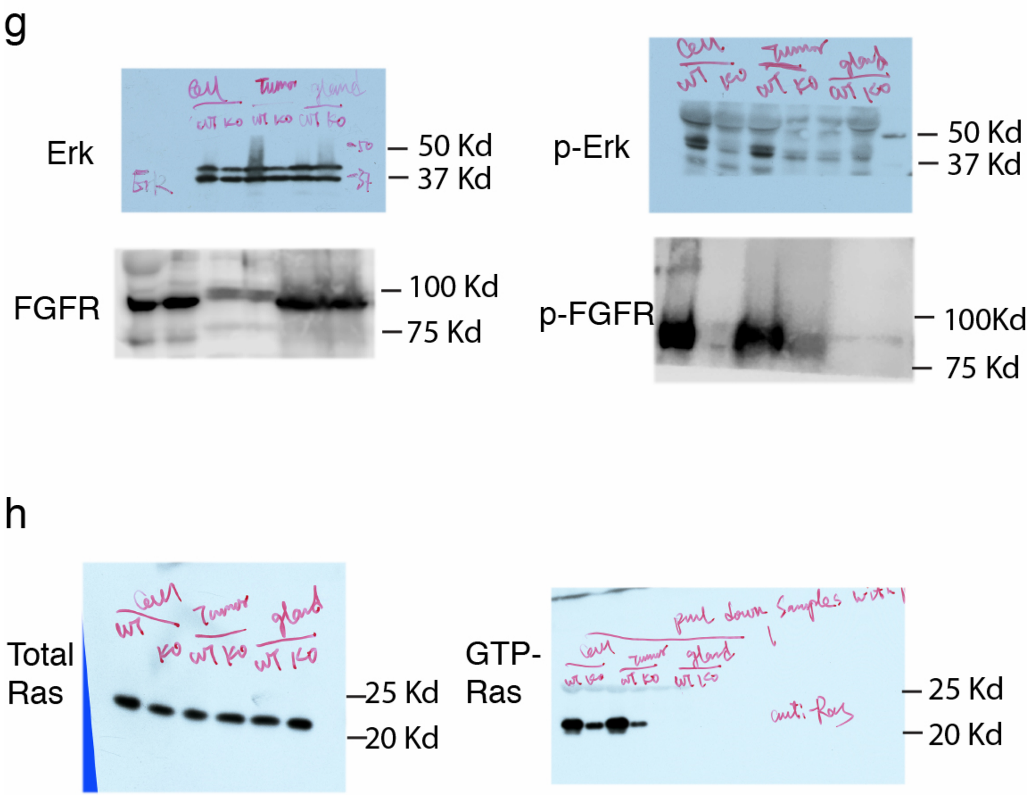

Supplementary Figure 9. Full scans of key immunoblots of Figure 3g and h.

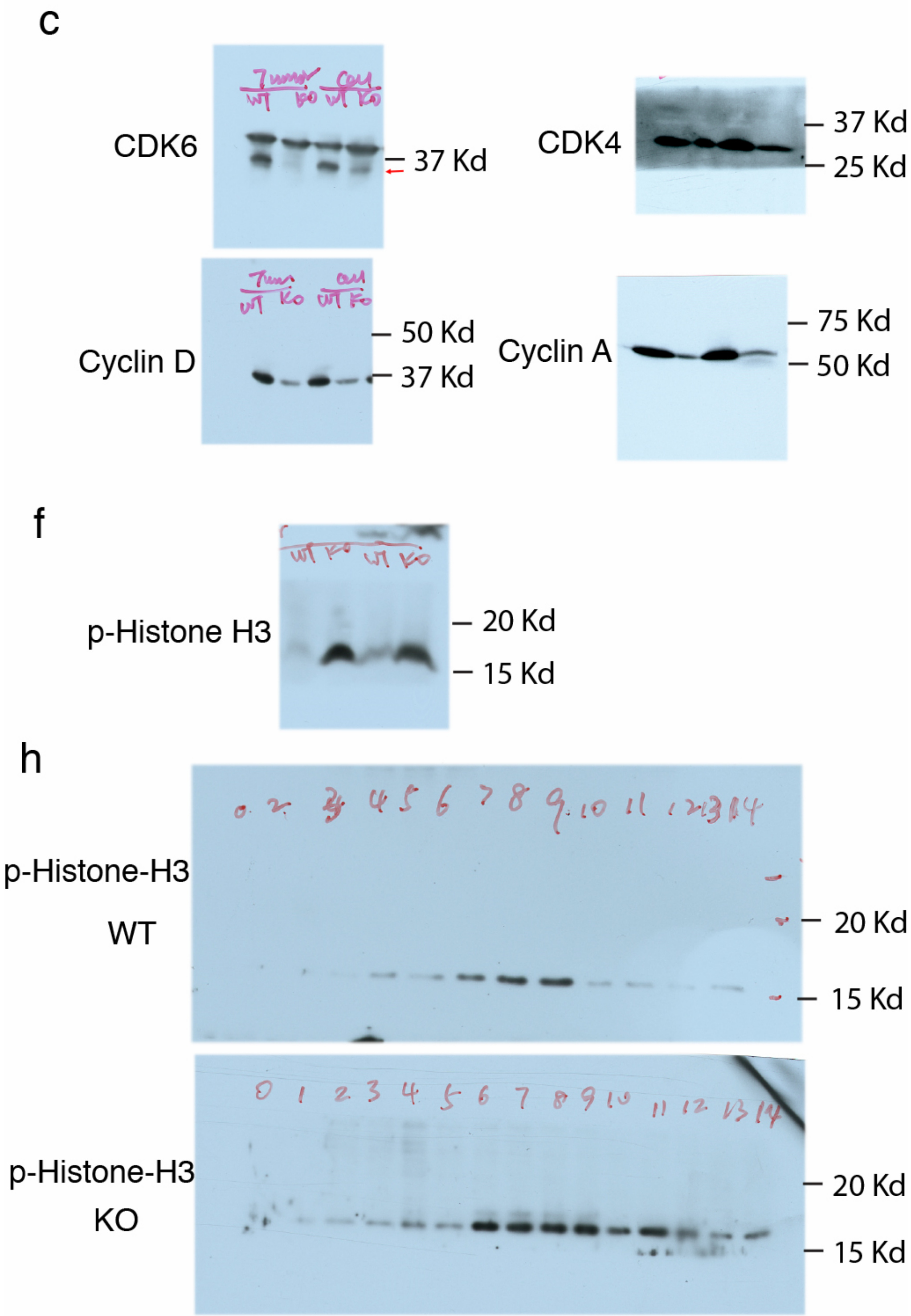

Supplementary Figure 10. Full scans of key immunoblots of Figure 4c, f, and h.

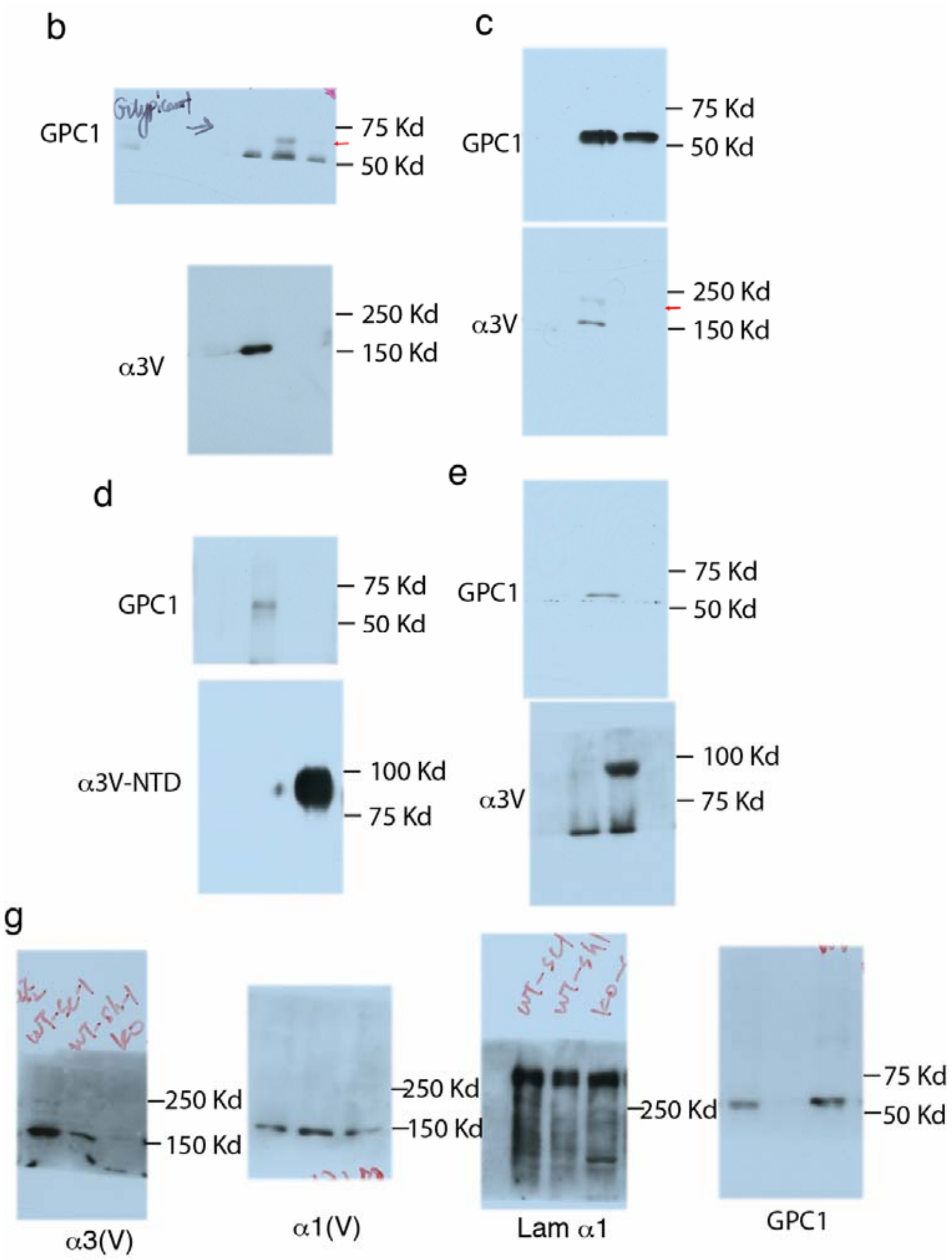

Supplementary Figure 11. Full scans of key immunoblots of Figure 5b - e and h.

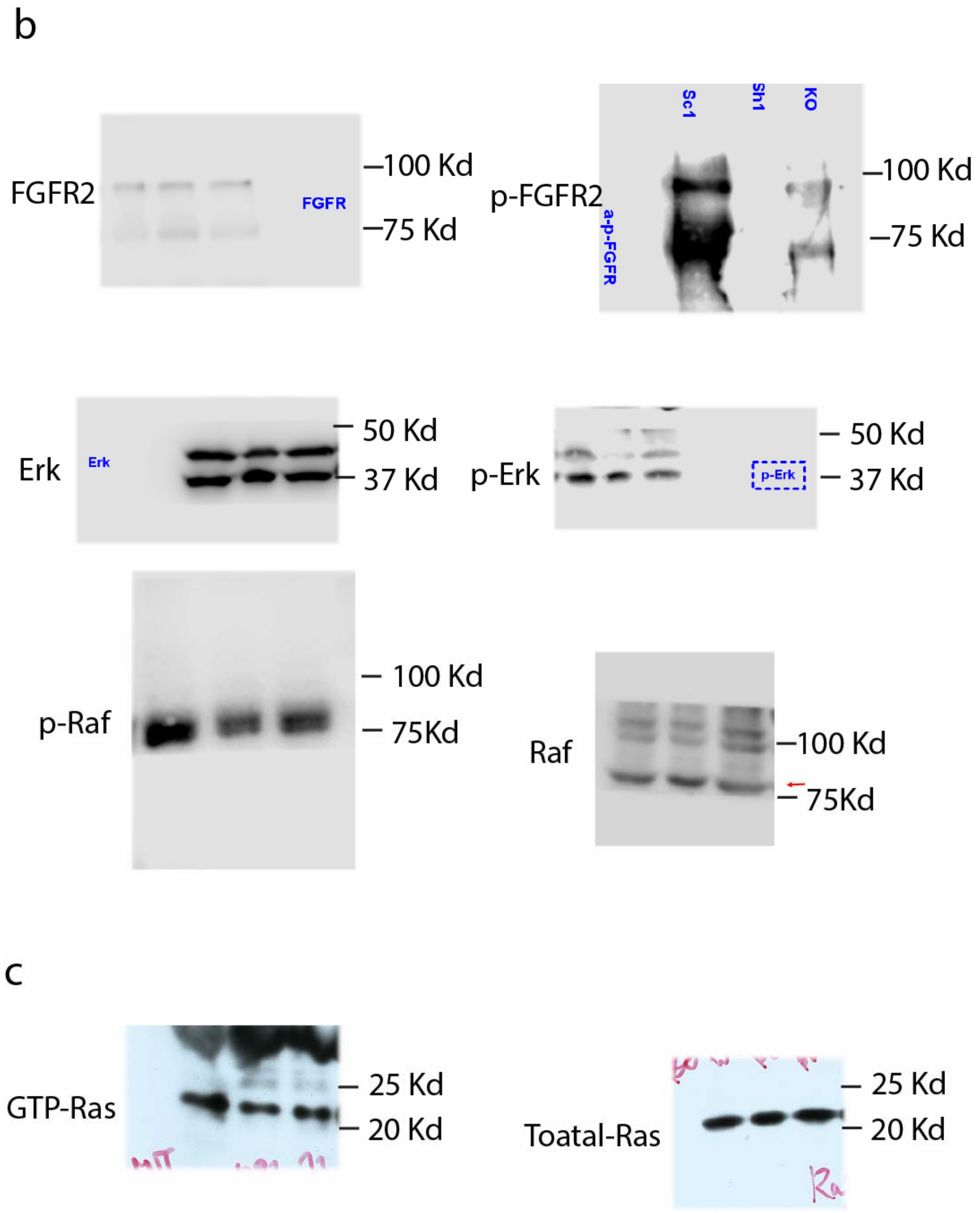

Supplementary Figure 12. Full scans of key immunoblots of Figure 6b and c.

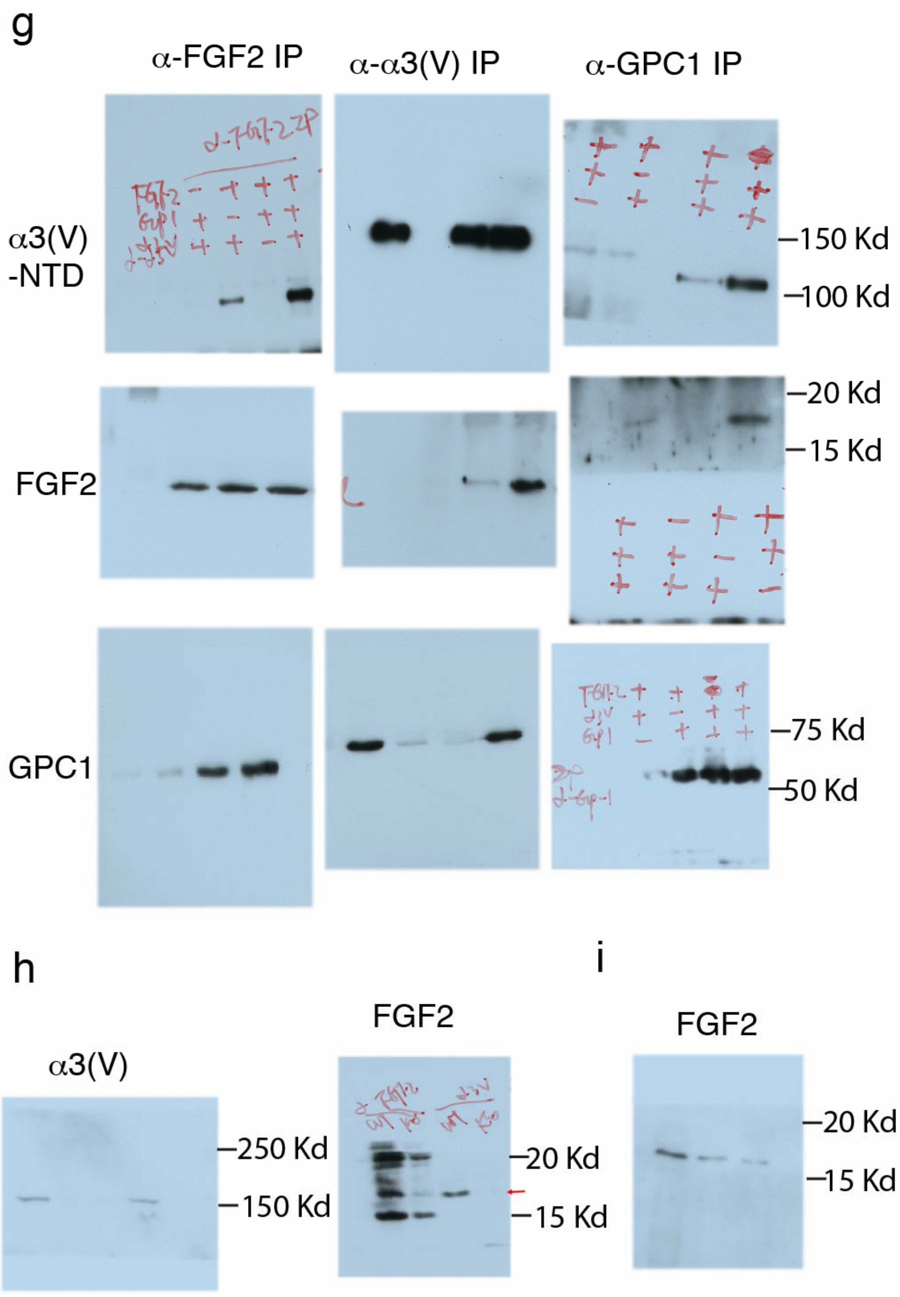

Supplementary Figure 13. Full scans of key immunoblots of Figure 7g - i.

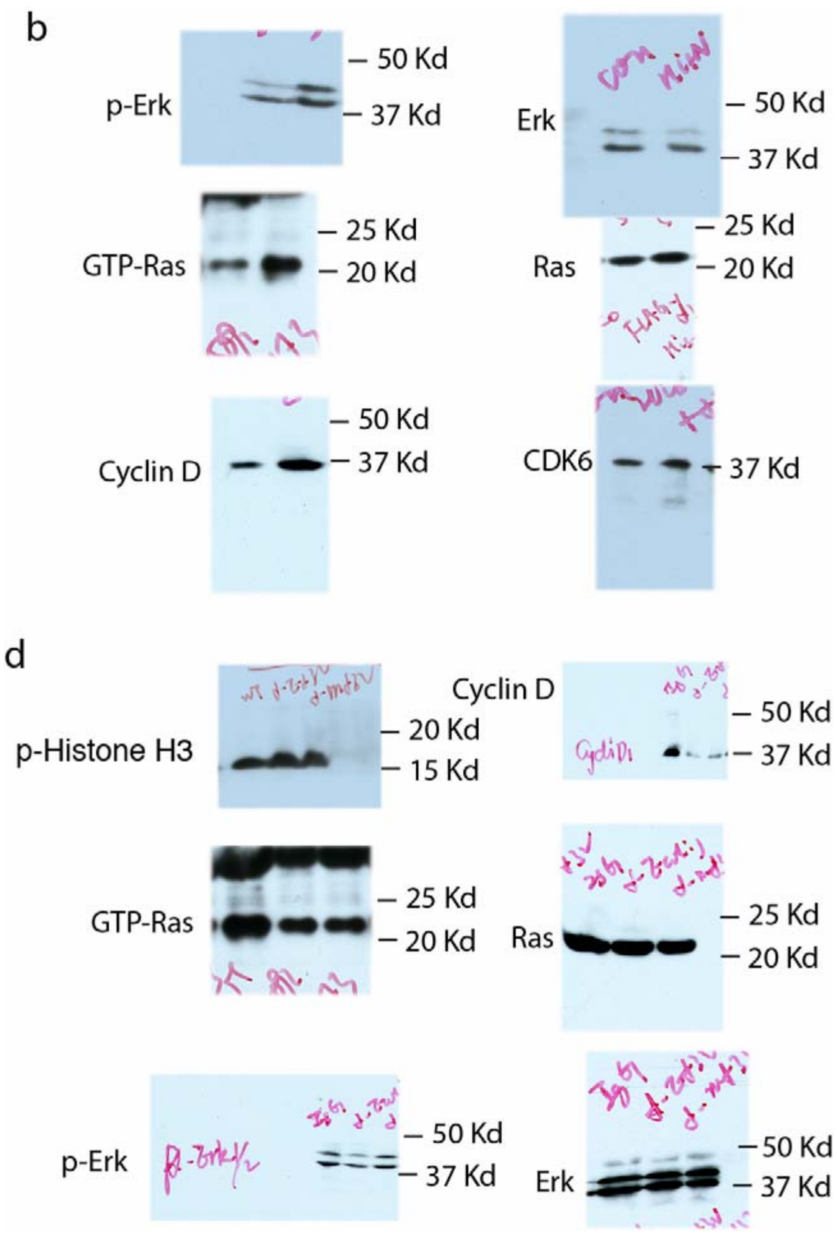

Supplementary Figure 14. Full scans of key immunoblots of Figure 8b and d.

Supplementary Table1: Length of cell cycle duration in WT/PyMT and KO/PyMT cells.

Genotype	G1	S	G2M	Total
WT	6	12	4	22*
KO	24	12	9	45

*All durations are given in hours.
